# Bioactivity and biocompatibility of bioceramic-based pulp capping materials in laboratory and animal models

**DOI:** 10.1007/s10856-025-06943-x

**Published:** 2025-10-14

**Authors:** Rafiqul Islam, Md. Refat Readul Islam, Kenta Tsuchiya, Yu Toida, Hidehiko Sano, Monica Yamauti, Hany Mohamed Aly Ahmed, Atsushi Tomokiyo

**Affiliations:** 1 Department of Restorative Dentistry, Faculty of Dental Medicine, Hokkaido University, Hokkaido, Japan; 2 Department of Oral Biology, School of Dental Medicine, University at Buffalo, The State University of New York, Buffalo, NY USA; 3 Department of Biomedical and Applied Science, School of Dentistry, Indiana University, Indianapolis, IN USA; 4 Department of Restorative Dentistry, Faculty of Dentistry, Universiti Malaya, Kuala Lumpur, Malaysia

**Keywords:** Bioactivity, Biocompatibility, Bioceramic material, Direct pulp capping, Mineral trioxide aggregate

## Abstract

Recently, premix bioceramic-based pulp capping (BPC) materials have been introduced with desirable properties. However, data regarding the bioactivity and biocompatibility of these materials remain limited. This present study aimed to evaluate the bioactivity and biocompatibility of BPC materials. This study consisted of five experimental groups: Bio C Repair (BCR); BG Multi (BGM); Well pulp ST (WPS); ProRoot MTA (PMTA); and a no capping (NC) group. Measurement of pH, calcium ion (Ca^2+^) release, and a bioactivity test were performed. An in vivo experiment was conducted on maxillary first molars of Wistar rats with exposed pulp, and pulpal responses were assessed at 1, 3, 7, and 28 days. Immunohistochemical expressions of Nestin, Osteopontin, and DMP-1 were performed. All materials exhibited alkaline pH. BCR exhibited the highest Ca^2+^ release (p < 0.05). PMTA, BCR, and WPS produced well-formed calcium phosphate depositions. On day 1, BCR, BGM and NC groups showed no to mild inflammatory responses (p < 0.05). On day 3, mild to moderate inflammatory responses was observed in all groups except for the NC group. On day 7, BCR and WPS groups exhibited no to mild inflammatory responses, along with the mineralized tissue layer formation (MTF). On day 28, no inflammatory responses were observed in the BCR, BGM, and WPS groups. Complete and homogenous MTF was identified in all experimental groups except the NC groups. Variable expression of Nestin, Osteopontin, and DMP-1 was noted at different time points. This present study demonstrated that premix BPC materials exhibited favorable bioactivity and biocompatibility and may serve as potential substitutes for PMTA.

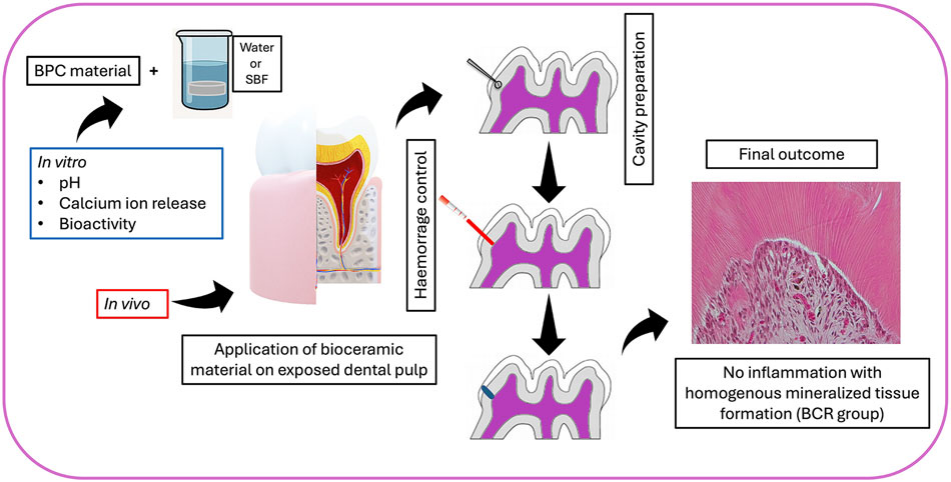

## Introduction

Recently, vital pulp therapy (VPT) has regained popularity for promoting dentin pulp regeneration [1]. For decades, calcium hydroxide [Ca(OH)_2_] was the material of choice for VPT, especially for direct pulp capping (DPC) treatment [2]. However, defects in the newly formed mineralized tissue and pulp necrosis, particularly in the long term, remain a concern [3]. As an alternative to CH, tooth-colored ProRoot mineral trioxide aggregate (PMTA; Dentsply Sirona, Charlotte, NC, USA) has gained popularity for DPC due to its ability to form a thicker dentinal bridge with favorable pulpal inflammation [4, 5]. Despite having these advantageous properties, handling difficulties, long setting time, and discoloration of the tooth are the major drawbacks of using PMTA [2, 6]. Therefore, researchers have focused on developing premix bioceramic-based pulp capping (BPC) material to address the drawbacks of PMTA [7, 8].

Premix BPC materials have gained attention due to their favorable physicochemical properties, such as convenient handling properties and shorter setting time, as well as prominent bioactivity and biocompatibility, which are key features necessary for successful VPT outcomes compared to PMTA [3, 9]. Additionally, these BPC materials have shown promise, particularly in their ability to release calcium ions (Ca^2+^) and promote an alkaline environment conductive to pulpal healing and the ability to induce homogenous mineralized tissue formation (MTF) [10]. Recently, new premix BPC has been introduced in a threaded syringe, offering better handling and insertion properties with a ready-to-use approach [11]. Nevertheless, there is a lack of studies evaluating the bioactivity and biocompatibility of newly premixed BPC materials, particularly in relation to their physicochemical properties and biological responses.

Although premix BPC materials have been increasingly adopted by clinicians, there is still limited knowledge regarding bioactivity and biocompatibility [12]. While several studies have investigated the properties of individual BPC materials, there is a lack of comparative preclinical-based study models that investigate multiple premix BPC materials [8, 13, 14]. To address this gap, the current study selected three distinct premixed BPC materials based on their different compositions, which are Bio C Repair™, Nishika Canal Sealer BG Multi™, and Well pulp ST™. These materials were chosen because they are widely available in several international markets and are increasingly used in clinical practice. Bio C Repair™ was chosen as it is a calcium silicate-based material and ready-to-use putty consistency, which is designed to improve upon the difficult handling and long setting time of MTA. On the other hand, Nishika Canal Sealer BG Multi™ is based on calcium silicate bioactive glass with Ca(OH)_2_ powder and magnesium oxide, which represents a different strategy to achieve bioactivity. Well, Pulp ST™ incorporates calcium alumino-silicate compounds with a fast-setting mechanism, which aimed at better clinical outcome. However, there is currently limited scientific data on the bioactivity and biocompatibility of these premixed BPC materials. Furthermore, physical properties such as ion release behavior, pH value, and potential in vitro assessment of bioactivity in a biologically simulated environment are important aspects that require additional investigation. This knowledge is crucial since differences in bioactivity and cellular responses within various materials can greatly affect the success and durability of VPT [15, 16].

Therefore, the current research aimed to evaluate the bioactivity in vitro and biocompatibility in vivo of the commercially available premix BPC capping materials that are readily available on the market. Additionally, physicochemical properties such as pH value and Ca^2+^ release were also evaluated. The null hypothesis was that there is no difference between the tested materials regarding pH, Ca^2+^ release ability, pulpal inflammation, and MTF.

## Materials and methods

### Experimental materials

The following experimental materials were included: commercially available premix BPC material, Bio C Repair™ (BCR; Angelus, Londrina, PR, Brazil), Nishika canal sealer BG Multi™ (BGM; Nishika, Shimonoseki, Japan), Well pulp ST™ (WPS; Vericom, Gangwon-Do, Korea), positive control—ProRoot MTA™ (PMTA; Dentsply Sirona), and negative control (NC)—non-bioactive restoration, restored with Super Bond™ (Sun Medical, Shiga, Japan). The compositions of the evaluated materials are shown in Table 1.

**Table 1:** Compositions of the materials used in this study

Materials	Manufacturers	Composition
ProRoot MTA™ (tooth coloured) (PMTA)	Dentsply Sirona, Charlotte, NC, USA	Tricalcium silicate, Dicalcium silicate, Tricalcium aluminate, Bismuth oxide, Gypsum
Bio-C Repair™ (BCR)	Angelus, Londrina, PR, Brazil	Calcium Silicate, Calcium oxide, Zirconium oxide, Silicon dioxide, Iron Oxide, and Dispersing agent
BG Multi™ (BGM)	Nishika, Shimonoseki, Japan	Paste A: Bismuth subcarbonate, fatty acids, silicon dioxide Paste B: Calcium silicate glass, Magnesium oxide, Purified water, Silicon dioxide Powder: Calicum silicate glass, Calcium hydroxide
Well Pulp St™ (WPS)	Vericom, Gangwon-Do, Korea	Calcium carbonate Silicon dioxide, Aluminum oxide, Zirconium dioxide, thickener, and others
Super- Bond™	Sun Medical, Shiga, Japan	Initiator- TBB, Monomer-MMA, 4-META Powder-PMMA powder

_*4- META* 4- methacryloyloxyethy trimellitate anhydride, *PMMA* polymethyl methacrylate, *MMA* methyl methacrylate, *TBB* tributylborane._

For in vitro testing, sample size calculation was performed using G*Power 3.1 software. Based on a one-way ANOVA design with four groups, an assumed effect size of 0.4, *α* = 0.05, and a power of 0.95, the total required sample size was calculated to be 112. To account for potential variation and improve reliability a 10% increase was applied, resulting in a final sample size of 124 specimens. Of these, 100 samples were used for pH and Ca^2+^ release measurements, while 24 samples were allocated for in vitro bioactivity assessment.

### pH and Ca^2+^ release analysis

A total of 100 discs of the four tested materials were prepared. Stainless steel ring molds with an internal diameter of 10 mm and a height of 1 mm were used for sample preparation. The molds were placed on a glass plate and filled to slight excess with the mixed materials. The same operator mixed all materials in accordance with the manufacturers’ instructions. After filling the molds, another glass plate covered with a mylar strip was placed on top of the molds, exerting a light pressure in order to remove any excess. All samples were left to set for 24 h in an incubator at 37 °C and 100% humidity. After 24 h, the specimens of each material were individually placed in polyethylene tubes, containing 10 mL of distilled water. Specimens were randomly allocated to the experimental groups using simple randomization. The samples (*n* = 5) were stored at 37 °C with an incubation period of 0 h (within 5 min), 1, 3, 7, and 28 days. The immersion distilled water was collected after each incubation period to analyze the pH value, which was measured by a digital pH meter (HM-25R, Tao DKK, Tokyo, Japan) equipped with a glass electrode. Before performing the pH analysis, the pH meter was calibrated. Briefly, the electrode was placed in the pH 7 buffer solution and left for about 1 min until the reading stabilizes at 7.0. After that, the electrode was rinsed with demineralized water, then placed in the pH 4 buffer solution, repeating the process until reliable readings were obtained. The same solution from the pH analysis was used to determine the Ca^2+^ release ability. The amount of Ca^2+^ released from each material was measured by inductively coupled plasma atomic emission spectroscopy (Agilent 5900 ICP-OES AP-100360, Agilent, Tokyo, Japan).

### Bioactivity testing

The bioactivity of the material groups was tested by assessing the precipitation on the surface of the material disc after their immersion in simulated body fluid (SBF). SBF was prepared by following the previously described method [17]. Each disc (*n* = 3 for each group) 10 mm in diameter and 1 mm in think, was prepared. The prepared samples were subsequently immersed in 10 mL of SBF solution within a flat-bottomed plastic vial container. The use of a flat-bottomed container ensures maximum surface contact between the samples and the SBF solution. The samples underwent immersion in 10 mL of SBF solution for a period of 28 days maintained at 37 °C in an incubator. This temperature closely resembles that of the human body, ensuring that the experimental environment closely mimic physiological conditions. Therefore, the SBF solution was replenished every 7 days throughout the 28-day immersion period. The SBF solution was prepared immediately before the beginning of each immersion period. This periodic replacement helps to maintain the chemical balance of the solution, ensuring that it remains conducive to apatite formation throughout the test. After 28 days, the discs were dried by placing the specimens in the incubator at 37 °C for 24 h and then positioned directly onto the stub for microscopic evaluation of the material surface. As a negative control, discs were prepared (*n* = 3 each) as describe above and immersed in the distilled water for 28 days and then dried by placing the specimens in the incubator at 37 °C for another 24 h. The bioactivity was assessed using a scanning electron microscope (SEM) (JSM-6510LA, Jeol, Tokyo, Japan) to determine the morphology of the formed deposits. Elemental analysis was performed using Energy-dispersive X-ray spectroscopy (EDX) (JSM-6510LA). During imaging, samples were focused on areas where mineral precipitates were visually evident. The working distance was maintained at approximately 10 mm, and the acceleration voltage was set to 15 kV with magnification at ×1500. All samples were coated with a thin layer of gold to ensure conductivity. During the EDX analysis, each specimen was assessed in six different random spot areas to determine the Ca/P ratio of the crystals formed [18].

### In vivo pulp capping model

**Fig. 1:**
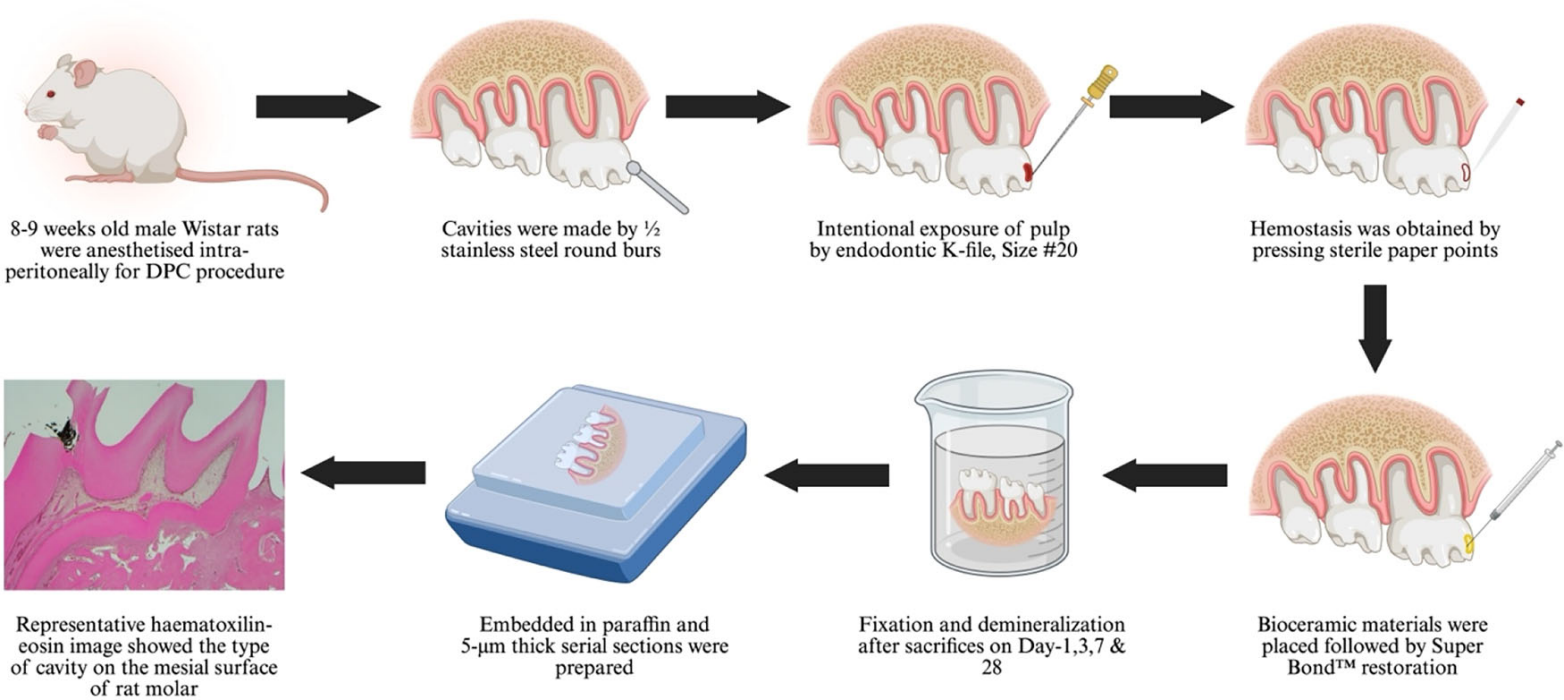
Schematic illustration of the in vivo experimental procedure

This in vivo experiment follows the Preferred Reporting Items for Animal Studies in Endodontology (PRIASE) 2021 guidelines [19]. This study was approved by the institutional ethical committee as well as by the committee for laboratory animals and breeding faculty in Hokkaido University (Ref. 21-0055) and performed according to animal care standards. The detailed procedure of the in vivo experiment is illustrated in Fig. 1.

Sixty Wistar rats obtained from Hokuto (Hokkaido, Japan), aged between 8 and 9 weeks and weighing between 220 and 300 g, were used for this study. The rats were fed by qualified animal caretakers in a sterile laboratory environment with a 12-h light/dark cycle at a suitable indoor temperature and humidity in the ranges of 20–26 °C and 40%–70%, respectively.

The number of animals used in this study was minimized in accordance with the ARRIVE guidelines [20]. G*Power software was utilized to determine the sample size and perform the power analysis. Based on our preliminary study, with an assumed alpha level of 0.05, an effect size of 0.17, and a power of 95%, a minimum of 120 samples was required. Consequently, 60 male Wistar rats were obtained to provide the 120 samples needed for this study, with each rat’s maxillary first molar chosen for the experiment. Adhering to the ARRIVE guidelines, the treatment was conducted carefully, and no sample loss was recorded.

### DPC procedure

The rats were simultaneously randomized (simple randomization) following the ARRIVE guidelines and divided into five experimental groups as mentioned above. The DPC procedure in this study was based on the method as previously described [21]. All the surgical instruments were sterilized in an autoclave. After taking all aseptic precautions, the rats were anaesthetized using a combination of inhaled isoflurane (Fujifilm Wako Chemical, Osaka, Japan) and intraperitoneal injections of medetomidine (ZENOAQ, Fukushima, Japan), midazolam (Maruishi Pharmaceutical, Osaka, Japan), and butorphanol (Meiji Seika Pharma, Tokyo, Japan) at doses of 0.75, 2, and 2.5 mg/kg/body weight per rat, respectively.

The DPC procedures were performed under magnification of a dental operating microscope (3.5×) (Professional L, HEINE Optotechnik, Gilching, Germany) with an assistant ensuring a sterile environment. After obtaining anesthesia, the rat molar teeth were rinsed with physiological saline solution (Otsuka Pharmaceutical, Tokyo, Japan) and then disinfected with cotton-soaked 75% ethanol (Fujifilm Wako Pure Chemical Corporation). Using sterile stainless-steel round burs (1/2 size; Emil Lange Zahnbohrerfabrik e.K, Engelskirchen, Germany), cavities were prepared on the mesial surfaces of the maxillary first molars. The burs were replaced after every two cavity preparations within the same rat. Continuous irrigation with distilled water was ensured during cavitation to prevent heat-induced pulpal damage. Sterile, size 20, stainless-steel K-files (Mani, Tochigi, Japan) were used to expose the remaining thin dentine of the cavity and the pulp. Hemostasis was obtained by pressing sterile paper points (Morita, Tokyo, Japan) on the exposed pulp for at least 1 min. After controlling bleeding, the tested material was placed at the exposure site. All the materials were mixed and placed according to the manufacturer’s instructions. Subsequently, all the cavities were restored with Super Bond™, which was mixed using the brush-dip technique as per the manufacturer’s instructions. To minimize occlusal forces, the cusp tip of the opposite teeth was slightly trimmed to ensure it did not make contact.

Following the surgical procedure, the rats received analgesic treatment with 5 mg/kg body weight of Carprofen (Zoetis, Tokyo, Japan) subcutaneously every 24 h, starting immediately and continuing for 2 additional days. The rats were returned to their allocated cages for recovery from anesthesia. Subsequently, a certified animal caretaker assessed postoperative indicators of pain. During the observation period, abnormal sleep patterns, persistent rubbing or scratching at specific areas, and reduced consumption of water and food were monitored. Finally, the rats were sacrificed at four specific time intervals: 1, 3, 7, and 28 days, with six teeth allocated for each interval.

### Fixation and histological tissue preparation

First, the rats were euthanized by administrating an overdose of intraperitoneal injections of anesthetic solutions after each time interval. Next, the treated maxillary first molar and some portions of the maxilla were precisely dissected and immersed in 4% paraformaldehyde solution (Nacalai Tesque, Kyoto, Japan) at 4 °C for 24 h to facilitate fixation. The dissected specimens were then washed with running water for 6 h. After that, the specimens were kept in 10% ethylenediamine tetraacetic acid (EDTA; Fujifilm Wako Pure Chemical) at room temperature with a pH of 7.4 for 21 days. Following the demineralization procedure, the restorations performed with Super Bond™ were carefully removed from the cavities and then rinsed with running water for an additional 24 h. Subsequently, the specimens were dehydrated in ascending ethanol grades and were then removed by immersing in xylene. Finally, the specimens were embedded in paraffin, and sliding microtomes were used to prepare 5-μm thick serial sections in mesiodistal orientations. To investigate the pulpal response, the slides were numbered, and median sections were chosen for haematoxylin–eosin (H&E) staining. Histological features were evaluated according to the criteria used in previous studies [21, 22], presented in Table 2, with two observers who were not informed of the true nature and purpose of the study. Inflammatory cell response and MTF were evaluated using scores of 0 to 3.

**Table 2:** Criteria used for histological analysis of the pulps with DPC: inflammatory cell response and mineralized tissue formation

Score	Inflammatory cell response	Mineralized tissue formation
0	No: Absent or very few inflammatory cells	No: no mineralized tissue deposition
1	Mild: inflammatory cell infiltration was observed adjacent to the newly formed mineralized tissue extending up to one third of the root canal pulp tissue	Initial: only a slight layer of mineralized tissue deposition
2	Moderate: inflammatory cells infiltration was observed in the part of coronal pulp/up to two thirds of the root canal pulp tissue	Partial/incomplete: mineralized tissue barrier formation extending more than half of the exposure site but not completely closing it
3	Severe: all coronal pulp/more than two thirds of the root canal pulp tissue is infiltrated or necrotic	Complete: complete mineralized tissue barrier formation

### Immunohistochemical examination

In all specimens, sections were subjected to immunohistochemistry for the detection of Osteopontin, Nestin, and Dentin matrix protein-1 (DMP-1). All sections were deparaffinized in xylene and then rehydrated by immersion in a series of alcohol solutions with increasing concentration: 70%, 80%, 90%, and 100% ethanol. Afterward, a 3% hydrogen peroxide solution was applied for 10 min to block endogenous peroxidase activity. Each section was then blocked with a 1% bovine serum albumin (BSA; Sigma-Aldrich, Missouri, USA) solution for 15 min, followed by incubation with the primary antibodies: rabbit anti-osteopontin monoclonal antibody (dilution 1:800, Abcam, Cambridge, UK), rabbit anti-Nestin monoclonal antibody (dilution 1:2000, Abcam), and rabbit anti-DMP-1 polyclonal antibody (dilution 1:500, Takara Bio) at room temperature for 60 min. The sections were then incubated for 30 min at room temperature with secondary antibodies of 3,3-diaminobenzidine to detect antibody-localized antigens and counterstained with a haematoxylin stain solution.

The stained sections were observed under a light microscope (Nikon Eclipse Ci, Nikon, Tokyo, Japan) for inflammatory response and MTF. To evaluate the immunohistochemically stained samples, a qualitative analysis was adopted based on direct visual comparison of immunohistochemical staining patterns. The assessment involved examining the overall staining intensity, pattern, and distribution across tissue sections for all tested materials. The comparison was made by two observers amongst the authors who were blinded to the group assignments. Both observers qualitatively evaluated the immunoreaction intensity and noted a visually stronger immunoreaction by denser staining and a deeper brown colour.

### Statistical analysis

To determine whether time influenced the pH values and Ca^2+^ release of the pulp capping materials, an analysis of longitudinal data was performed using the *t*-test for paired data (*p* < 0.05) between different times of incubation. For histological analysis, the distribution of the data was analyzed by the Kolmogorov–Smirnov normality test. Due to a nonparametric data distribution, the Kruskal-Wallis followed by Mann–Whitney U test was used to determine the difference between groups in each observational period. Multiple comparison corrections were not applied as the pairwise tests were considered pre-planned comparisons based on specific hypotheses. Box plots were generated using the 95% confidence interval to indicate data distribution. Statistical analysis was performed using IBM SPSS statistics for Windows, version 28 (IBM, Chicago, IL, USA). A *p* value of <0.05 was considered statistically significant.

## Results

### pH measurement

**Fig. 2:**
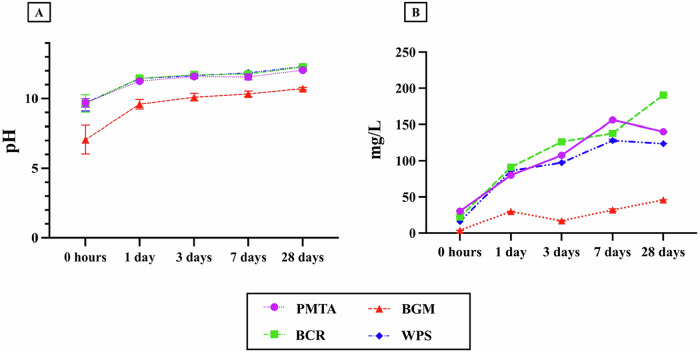
pH and Ca^2+^ release curves at 0 h, 1, 3, 7, and 28 days. Data are presented as mean ± standard deviation. **A** pH curves of the evaluated materials; **B** Ca^2+^ release curves of the evaluated materials. Abbreviations: PMTA ProoRoot MTA, BCR Bio C Repair, BGM BG Multi, WPS Well Pulp ST

All the values are presented as mean ± standard deviation. Immediately after immersion, PMTA (9.69 ± 0.30), BCR (9.65 ± 0.62), and WPS (9.69 ± 0.58) exhibited almost similar alkaline pH values, while the BGM value (7.06 ± 1.02) was significantly lower (*p* < 0.05), closer to a neutral pH (Fig. 2A). On day 1, all groups exhibited increased pH levels, with PMTA (11.24 ± 0.07), BCR (11.46 ± 0.13), and WPS (11.44 ± 0.08) values above 11, further establishing an alkaline environment. In contrast, BGM (9.59 ± 0.34) remained significantly lower than the other groups (*p* < 0.05). On day 3, PMTA (11.59 ± 0.16), BCR (11.72 ± 0.07), and WPS (11.64 ± 0.08) continued to rise gradually, maintaining a highly alkaline range. BGM (10.10 ± 0.27) also exhibited an upward trend, although its pH remained significantly lower compared to the other materials (*p* < 0.05). By day 7, PMTA (11.55 ± 0.20), BCR (11.76 ± 0.07), and WPS (11.85 ± 0.04) values stabilized near their peak alkaline levels, whereas BGM continued its upward trend to approximately 10.33 ± 0.19 (*p* < 0.05). On day 28, PMTA (12.04 ± 0.06), BCR (12.26 ± 0.04), and WPS (12.31 ± 0.05) achieved their highest pH values, with BCR and WPS maintaining slightly higher alkalinity than PMTA. BGM (10.72 ± 0.09) exhibited a slight increase but remained significantly lower than the other materials (*p* < 0.05). Overall, PMTA, BCR, and WPS followed similar alkaline trends, while BGM displayed lower initial and peak pH values.

### Ca^2+^ release analysis

Immediately after immersion (within 5 min), the calcium release values showed distinct differences among materials (Fig. 2B). PMTA exhibited the highest release at 30.37 ± 0.96 mg/L, followed by BCR at 22.21 ± 0.67 mg/L, and WPS at 15.99 ± 0.70 mg/L, while BGM had a notably lower value of 3.83 ± 0.22 mg/L (Fig. 2B). On day 1, calcium release increased across all groups, with BCR reaching the highest level at 91.11 ± 0.91 mg/L, followed closely by WPS at 86.45 ± 0.96 mg/L and PMTA at 80.14 ± 0.99 mg/L, while BGM showed an increase to 29.91 ± 0.37 mg/L. On day 3, PMTA and BCR maintained high calcium release values, with BCR reaching 126.38 ± 1.25 mg/L and PMTA at 107.60 ± 1.33 mg/L. In contrast, BGM exhibited a lower release at 16.90 ± 0.85 mg/L, while WPS showed an increasing trend at 97.44 ± 0.49 mg/L. On day 7, the calcium release continued to increase for PMTA, reaching 156.09 ± 1.08 mg/L, while WPS and BCR also exhibited high values of 128.05 ± 2.01 mg/L and 138.08 ± 1.07 mg/L, respectively. However, BGM remained relatively low at 31.89 ± 1.73 mg/L. By day 28, the calcium release values shifted slightly, with BCR reaching its peak at 190.49 ± 0.29 mg/L, followed by PMTA at 140.37 ± 0.16 mg/L, and WPS at 123.70 ± 0.06 mg/L, whereas BGM maintained a lower value of 46.03 ± 0.09 mg/L. Significant differences were observed between all the materials (*p* < 0.05) at all time frames.

### In vitro bioactivity testing

**Fig. 3:**
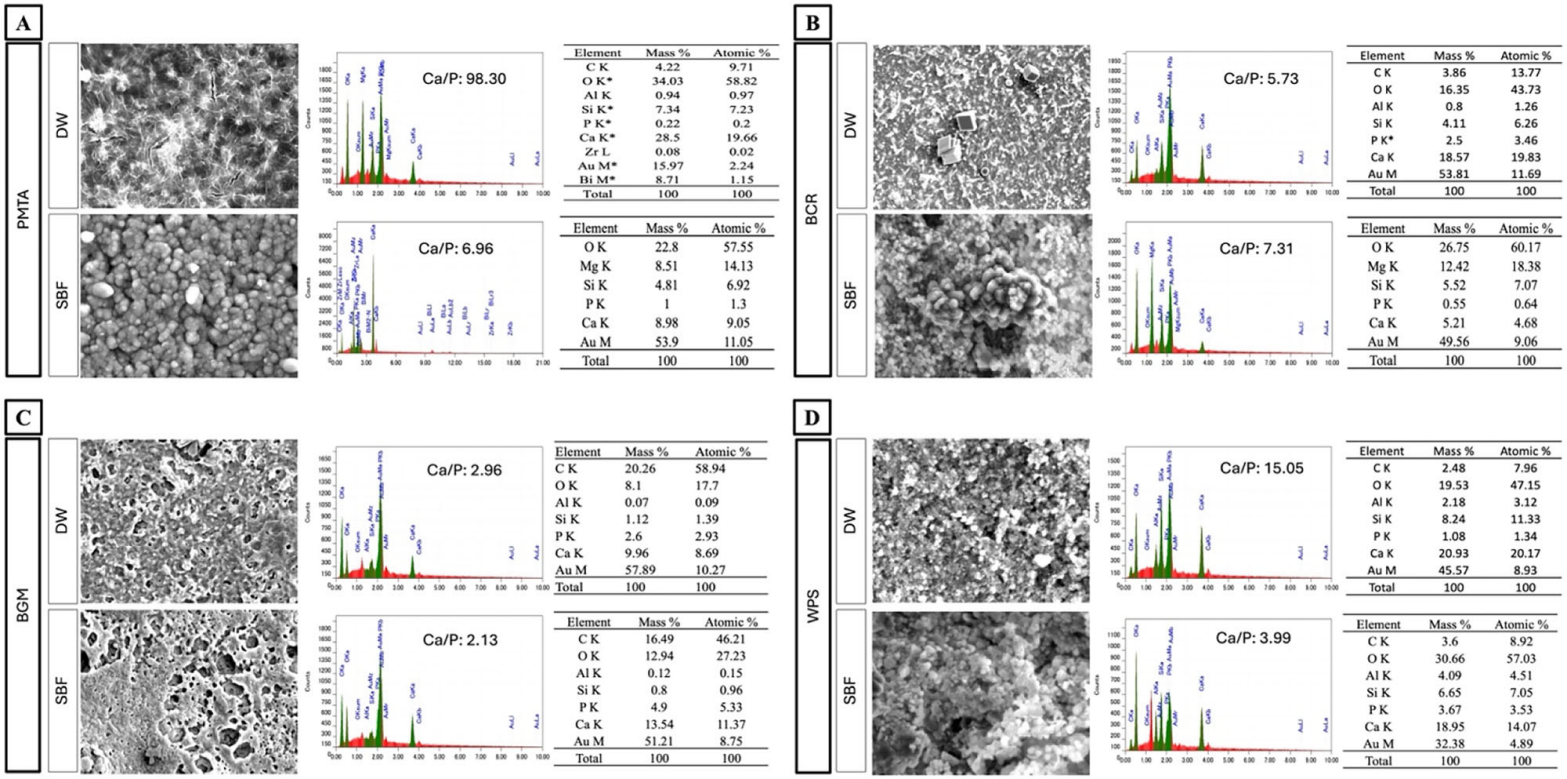
SEM and EDX analysis of the tested materials after 28 days of immersion in DW and SBF. **A** PMTA groups. DW immersion exhibited a dense matrix interspersed with crystalline structures, while SBF immersion led to granular surface structure; **B** BCR groups. They displayed a uniform morphology with visible filler-like microstructures in DW, which transformed into a granular mineralized structure in SBF; **C** BGM groups. They showed a bubble-like surface with matrix both in DW and SBF; **D** WPS groups. They had a granular morphology in DW, with increased mineral deposition in SBF. EDX spectra for all materials demonstrated enhanced calcium and phosphorus peaks in SBF, supporting their bioactivity and potential for MTF. Abbreviations: PMTA ProoRoot MTA, BCR Bio C Repair, BGM BG Multi, WPS Well Pulp ST, DW distilled water, SBF simulated body fluid, MTF mineralized tissue formation

All tested materials exhibited bioactive behavior after immersion in SBF for 28 days (Fig. 3). In PMTA group, SEM analysis revealed a granular surface structure with uniform deposition. In the BCR and WPS groups, the surface exhibited well-formed cluster of globular structures. In the BGM group, SEM images exhibited a surface with bubbles along with the matrix. EDX elemental analysis further identified peaks for calcium and phosphorus for all the materials. The calculated EDS Ca/P ratios were as follows: in the DW group PMTA: 98.30, BCR: 5.73, BGM: 2.96, and WPS: 15.05; and in the SBF group PMTA: 6.96, BCR: 7.31, BGM: 2.13, and WPS: 3.99.

On the other hand, the SEM micrographs of the set cement disc immersed in the distilled water as a control for all tested materials revealed distinct surface morphologies with no layer formation after 28 days (Fig. 3). In the PMTA group, the microstructure displayed a dense matrix interspersed with crystalline structures. In the BCR groups, the surface exhibited a relatively uniform morphology with visible filler-like microstructures. In the BGM group, the SEM images showed a surface with numerous voids. In the WPS group, the surface appeared granular with fine particles dispersed throughout. For all the groups, the EDX analysis showed prominent peaks for calcium, silicon, oxygen, and other elements.

### In vivo pulp capping model

**Fig. 4:**
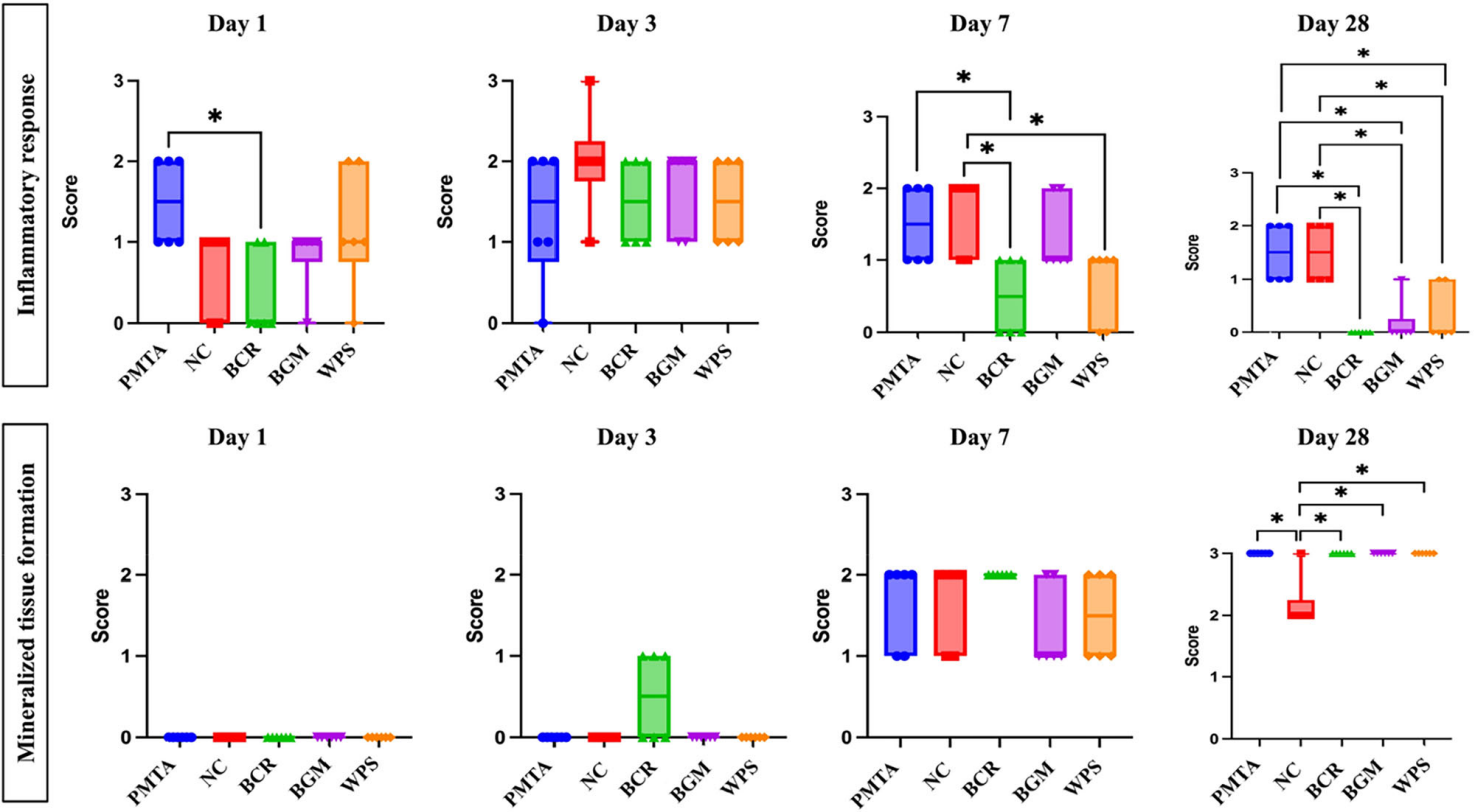
Box plot of data for inflammatory response and mineralized tissue formation at day 1, 3, 7, and 28. Asterisks indicate a significant difference between groups (*p* < 0.05). Abbreviations PMTA ProoRoot MTA, NC negative control, BCR Bio C Repair, BGM Nishika Canal Sealer BG Multi, WPS Well Pulp ST

The statistical analytical results of the inflammatory response and MTF are shown in Fig. 4.

#### Observation period on day 1

**Fig. 5:**
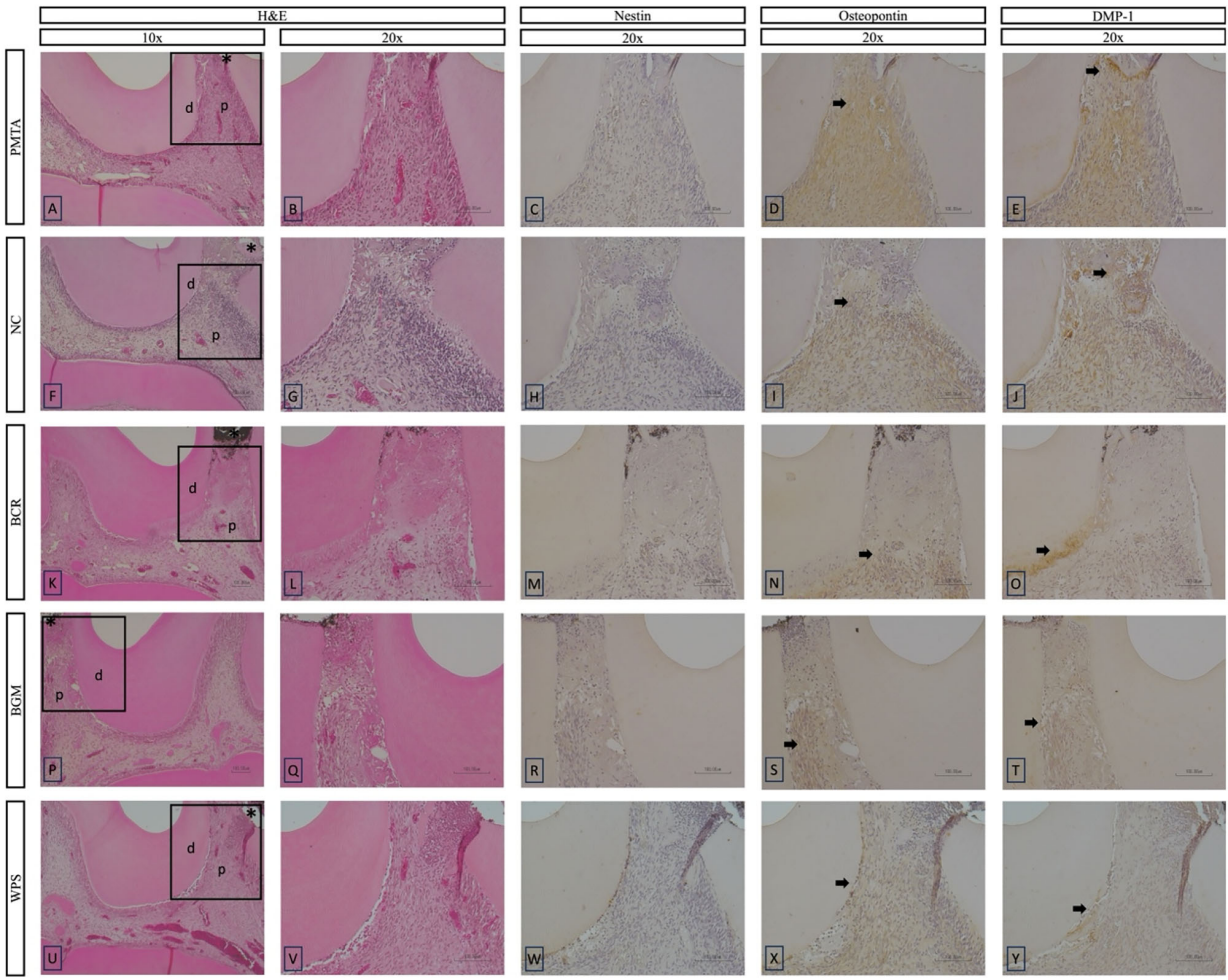
Representative histological HE and immunohistochemical images at day 1 of the tested materials at 10x and 20x magnifications. **B**–**E**, **G**–**J**, **L**–**O**, **Q**–**T**, and **V**–**Y** present a higher magnification of the areas (inset rectangles) in **A**, **F**, **K**, **P**, and **U**, respectively. **A**–**E**, PMTA group; **F**–**J**, NC group; **K**–**O**, BCR group; **P**–**T**, BGM group; **U**–**Y**, WPS group. HE staining (**A**, **B**, **F**, **G**, **K**, **L**, **P**, **Q**, **U**, **V**) shows pulpal tissue responses. Immunohistochemical staining highlights the expression of odontogenic markers: Nestin (**C**, **H**, **M**, **R**, **W**), Osteopontin (**D**, **I**, **N**, **S**, **X**), and DMP-1 (**E**, **J**, **O**, **T**, **Y**). Abbreviations: HE haematoxylin–eosin, PMTA ProoRoot MTA, NC negative control, BCR Bio C Repair, BGM Nishika Canal Sealer BG Multi, WPS Well Pulp ST, DMP-1 Dentin matrix protein-1, d dentin, p pulp tissue. Arrows indicate positively stained areas, and asterisks indicate pulp exposure areas

On day 1, inflammatory responses varied across groups. The PMTA and WPS groups exhibited mild to moderate inflammatory responses, while the NC, BCR, and BGM groups showed no to mild inflammatory responses (Figs. 4 and 5). Significant differences were observed between the PMTA group and the NC, BCR, and BGM groups (*p* < 0.05). For MTF, none of the groups showed any mineralization, indicating no detectable formation at this initial time point. Immunohistochemical staining (Fig. 5) for Nestin, Osteopontin, and DMP-1 revealed varying levels of expression among the groups. No evidence of Nestin immunoreactivity was observed adjacent to the exposure site in any of the groups. Osteopontin expression was positive at the exposure area in all the groups, with more prominent expression in the PMTA, NC, and BCR groups. The BGM and WPS groups showed lower Osteopontin expression. Positive expression of DMP-1 was observed at the exposure area in the PMTA and NC groups, whereas in the other groups, DMP-1 expression was detected further away from the exposure area.

#### Observation period on day 3

**Fig. 6:**
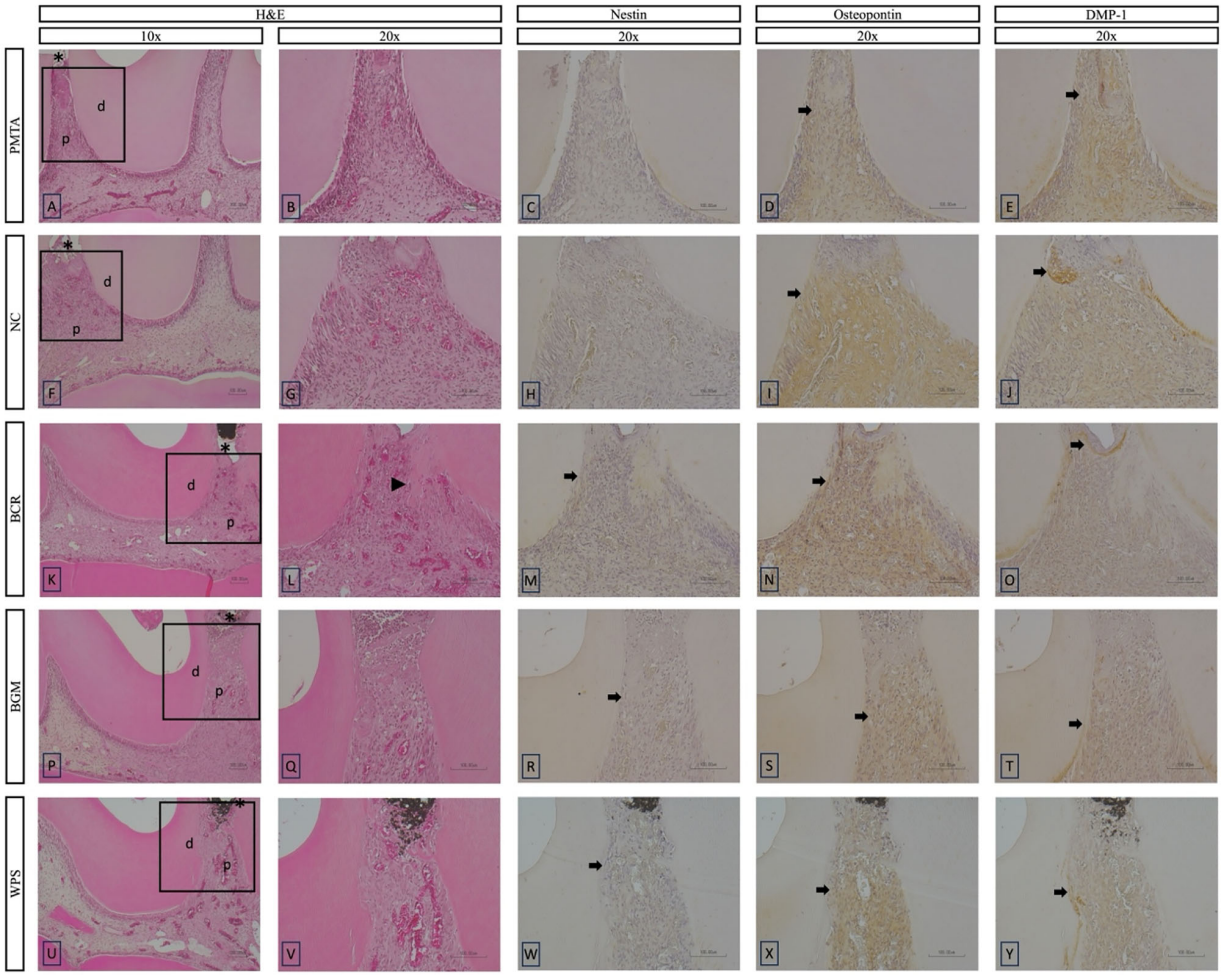
Representative histological HE and immunohistochemical images at day 3 of the tested materials at 10x and 20x magnifications. **B**–**E**, **G**–**J**, **L**–**O**, **Q**–**T**, and **V**–**Y** present a higher magnification of the areas (inset rectangles) in **A**, **F**, **K**, **P** and **U**, respectively. **A**–**E**, PMTA group; **F**–**J**, NC group; **K**-**O**, BCR group; **P**–**T**, BGM group; **U**–**Y**, WPS group. HE staining shows pulpal tissue responses (**A**, **B**, **F**, **G**, **K**, **L**, **P**, **Q**, **U**, **V**). Immunohistochemical staining highlights the expression of odontogenic markers: Nestin (**C**, **H**, **M**, **R**, **W**), Osteopontin (**D**, **I**, **N**, **S**, **X**), and DMP-1 (**E**, **J**, **O**, **T**, **Y**). Abbreviations: HE haematoxylin–eosin, PMTA ProoRoot MTA, NC negative control, BCR Bio C Repair, BGM Nishika Canal Sealer BG Multi, WPS Well Pulp ST, DMP-1 Dentin matrix protein-1, d dentin, p pulp tissue. Arrows indicate positively stained areas, asterisks indicate pulp exposure areas, and a black arrowhead indicates a thin layer of mineralization

On day 3, all the groups exhibited mild to moderate inflammatory responses except for the NC group, which showed a moderate inflammatory response (Figs. 4 and 6). In terms of MTF, none of the groups showed any mineralization except the BCR group, which showed a thin layer of mineralization. Mild Nestin expression was observed at the exposure area in the BCR, BGM, and WPS groups (Fig. 6). Osteopontin expression was positive at the exposure area in all the groups, with strong expression observed in the NC, BCR, and WPS groups. The PMTA and BGM groups showed lower Osteopontin expression. Positive expression of DMP-1 was observed at the exposure area in the NC and BCR groups.

#### Observation period on day 7

**Fig. 7:**
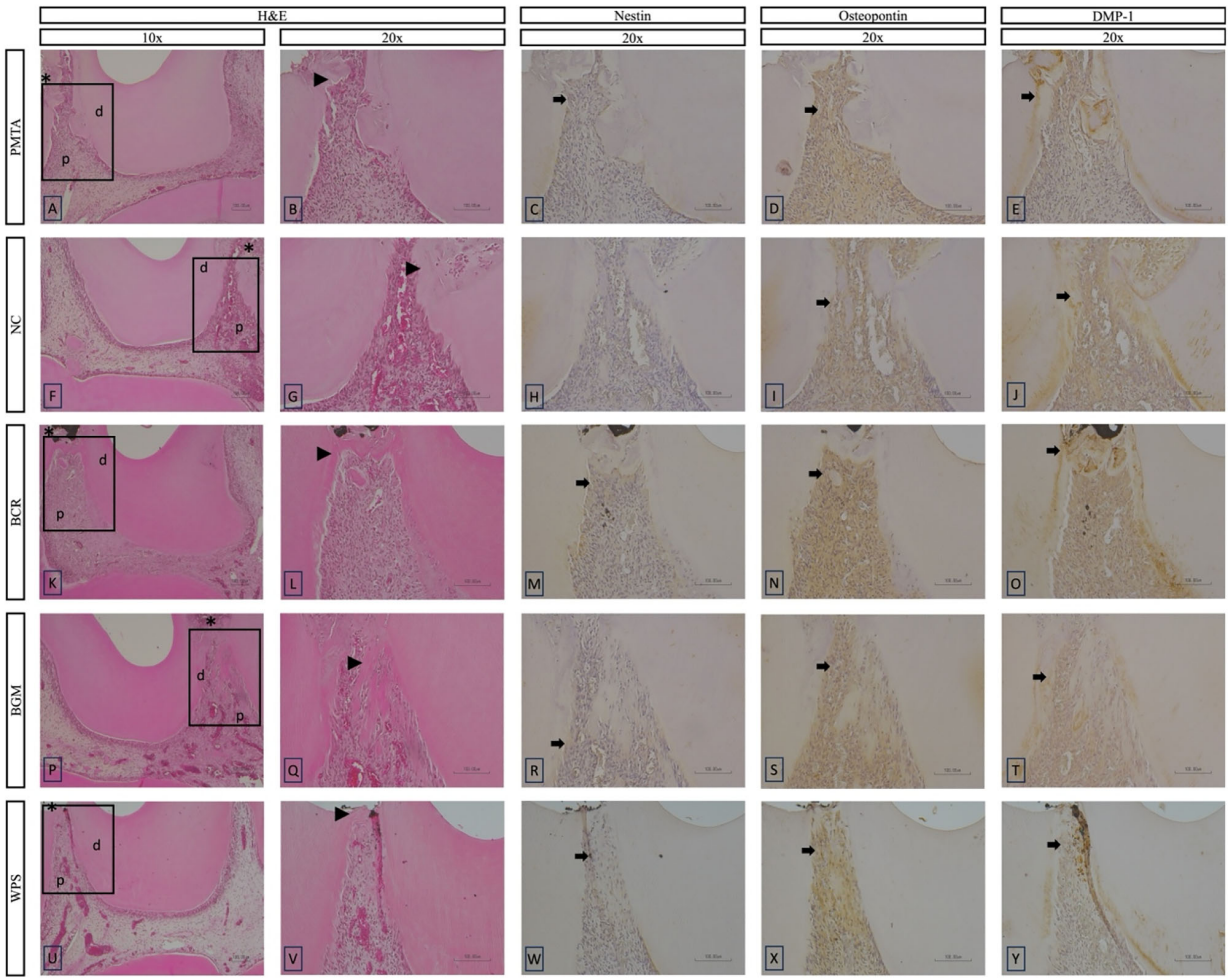
Representative histological HE and immunohistochemical images at day 7 of the tested materials at 10x and 20x magnifications. **B**–**E**, **G**–**J**, **L**–**O**, **Q**–**T,** and **V**–**Y** present a higher magnification of the areas (inset rectangles) in **A**, **F**, **K**, **P**, and **U**, respectively. **A**–**E**, PMTA group; **F**–**J**, NC group; **K**–**O**, BCR group; **P**–**T**, BGM group; **U**–**Y**, WPS group. HE staining shows pulpal tissue responses (**A**, **B**, **F**, **G**, **K**, **L**, **P**, **Q**, **U**, **V**). Immunohistochemical staining highlights the expression of odontogenic markers: Nestin (**C**, **H**, **M**, **R**, **W**), Osteopontin (**D**, **I**, **N**, **S**, **X**), and DMP-1 (**E**, **J**, **O**, **T**, **Y**). Abbreviations: HE haematoxylin–eosin, PMTA ProoRoot MTA, NC negative control, BCR Bio C Repair, BGM Nishika Canal Sealer BG Multi, WPS Well Pulp ST, DMP-1 Dentin matrix protein-1, d dentin, p pulp tissue. Arrows indicate positively stained areas, asterisks indicate pulp exposure areas, and black arrowheads indicate thin layers of mineralization

On day 7, no to mild inflammatory responses were observed at the exposure area in the BCR and WPS groups, whereas mild to moderate inflammatory responses were observed in the PMTA, NC, and BGM groups (Figs. 4 and 7). Significant differences were observed between the BCR group and the PMTA, NC, and BGM groups (*p* < 0.05). In terms of MTF, all the groups showed a layer of MTF at the exposure area, with a homogenous MTF seen in the PMTA and BCR groups. In terms of immunohistochemical analysis, positive expressions of Nestin were observed at the exposure area in the PMTA, BCR, and WPS groups, whereas the expression in the BGM group was detected further away from the exposure area (Fig. 7). Osteopontin expression was positive at the exposure area in all the groups, with strong expression observed in the BCR, BGM, and WPS groups. Positive expression of DMP-1 was observed at the exposure area in all the groups.

#### Observation period on day 28

**Fig. 8:**
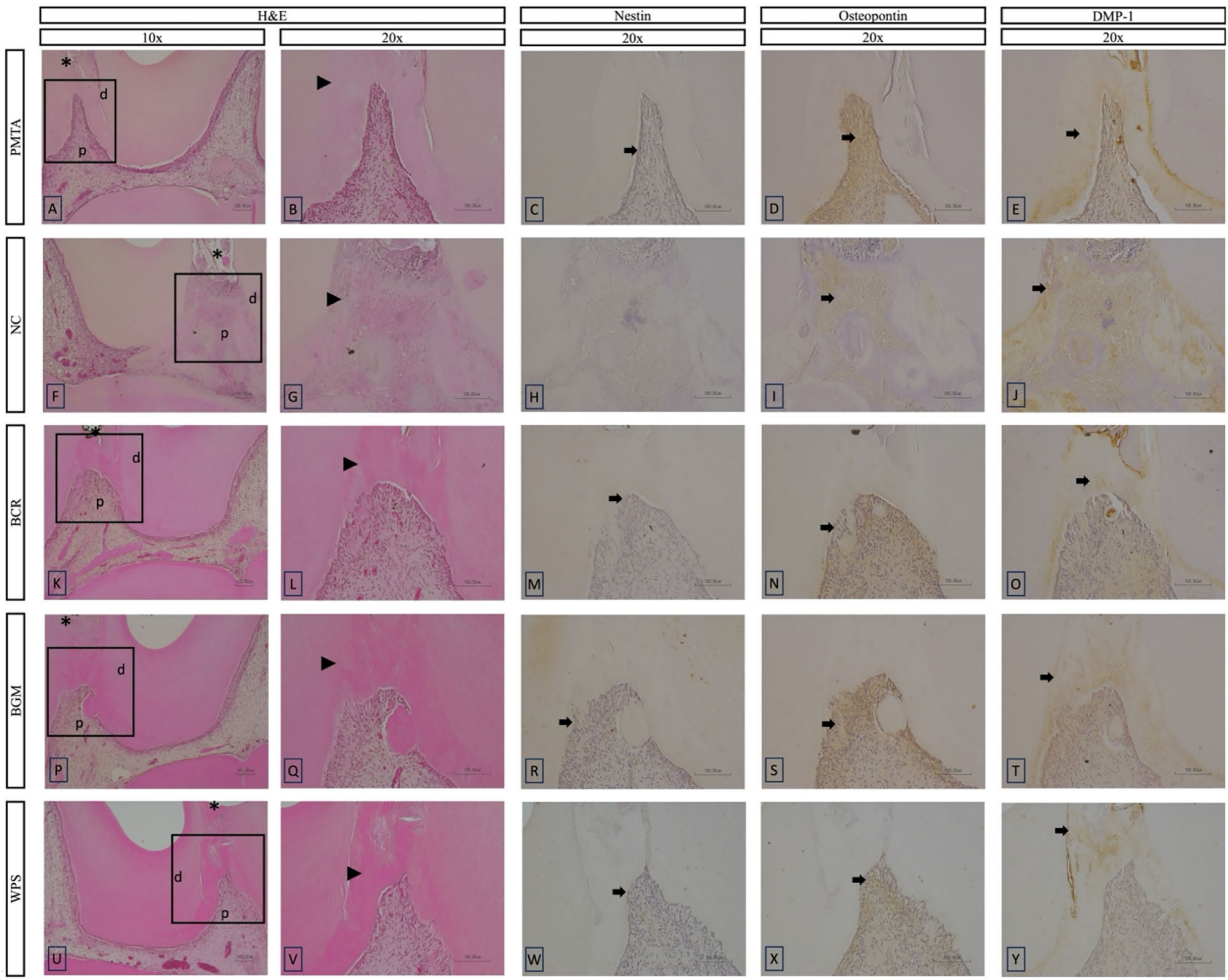
Representative histological HE and immunohistochemical images at day 28 of the tested materials at 10x and 20x magnifications. **B**–**E**, **G**–**J**, **L**–**O**, **Q**–**T**, and **V**–**Y** present a higher magnification of the areas (inset rectangles) in **A**, **F**, **K**, **P**, and **U**, respectively. **A**–**E**, PMTA group; **F**–**J**, NC group; **K**–**O**, BCR group; **P**–**T**, BGM group; **U**–**Y**, WPS group. HE staining shows pulpal tissue responses (**A**, **B**, **F**, **G**, **K**, **L**, **P**, **Q**, **U**, **V**). Immunohistochemical staining highlights the expression of odontogenic markers: Nestin (**C**, **H**, **M**, **R**, **W**), Osteopontin (**D**, **I**, **N**, **S**, **X**), and DMP-1 (**E**, **J**, **O**, **T**, **Y**). Abbreviations: HE haematoxylin–eosin, PMTA ProoRoot MTA, NC negative control, BCR Bio C Repair, BGM Nishika Canal Sealer BG Multi, WPS Well Pulp ST, DMP-1 Dentin matrix protein-1, d dentine, p pulp tissue. Arrows indicate positively stained areas, asterisks indicate pulp exposure areas, black arrowheads indicate thin layers of mineralization, and a white arrowhead indicates a dentine tubule-like structure

On day 28, almost no inflammatory responses were observed in the BCR, BGM, and WPS groups, whereas mild to moderate inflammatory responses were observed in the PMTA and NC groups (Figs. 4 and 8). Significant differences were observed between the PMTA group and the BCR, BGM, and WPS groups, and between the NC group and the BCR, BGM, and WPS groups (*p* < 0.05). In terms of MTF, all the groups showed complete homogenous MTF except the NC groups, which showed incomplete MTF. In addition, homogenous dentine tubule-like structures were observed in the BCR group in MTF. Significant differences were observed between the NC group and other groups (*p* < 0.05). In terms of immunohistochemical analysis, positive expression of Nestin was observed just beneath the MTF in all the groups except the NC group (Fig. 8). Osteopontin expression was positive in all the groups, with mild expression observed in the NC group. Positive expression of Nestin were observed just beneath the MTF in all the groups except NC group. Positive expression of DMP-1 was observed within the MTF in all the groups.

## Discussion

In the present study, significant differences were observed in the pH values, Ca^2+^ release ability, pulpal inflammation, and MTF. Therefore, the null hypothesis was rejected for all study parameters. The pH values of all the tested materials possesses an alkaline environment. This result indicates their ability to promote biologically favorable MTF [23]. An alkaline environment has the ability to induce osteogenic potential and modulation of inflammation [24–26]. The pH observed in ProRoot MTA™ (PMTA) and Bio-C Repair™ (BCR) can be primarily attributed to the presence of calcium silicate phase, which undergoes hydration to produce Ca(OH)_2_. The release of hydroxide ion from Ca(OH)_2_ elevates the pH, which contributes to a highly alkaline environment. Although Well pulp ST™ (WPS) does not contain tricalcium silicate, its composition includes calcium carbonate, which may contribute to alkalinity through dissolution and ionic exchange in aqueous environment [27]. In addition, these findings align with previous studies that highlight the importance of high alkalinity in stimulating the mineralization process and neutralizing bacterial activity [28, 29]. On the other hand, Nishika Canal Sealer BG Multi™ (BGM) exhibited a significantly lower pH at all time intervals compared to other groups, but it still possessed an alkaline environment. This may be related to the formulation based on calcium silicate glass and the inclusion of magnesium oxide which tends to buffer the solution and limits the release of free hydroxide ions. Magnesium is an essential ion for bone metabolism and is known to participate in biomineralization. Inclusion of magnesium in bioceramic materials may influence physical properties and support the proliferation of dental pulp cells [30]. The presence of magnesium could have contributed to its favorable biocompatibility, as evidenced by the resolution of inflammation and complete MTF [30]. Previous studies demonstrated that an alkaline environment could enhance biomineralization and modulate inflammatory response, potentially contributing to favorable tissue repair [31, 32].

The release of Ca^2+^ is a crucial factor in maintaining the bioactivity of any DPC materials [33, 34]. In addition, Ca^2+^ release has been correlated with the biological properties of DPC materials, as it stimulates the differentiation potential of dental pulp cells and promotes mineralization, leading to hard tissue formation [35]. However, the release of Ca^2+^ depends on the composition of the mineral particles of DPC materials, which are responsible for water solubility and diffusion [36]. In this study, PMTA, BCR, and WPS exhibited the highest Ca^2+^ release over the observation period. These findings suggest that a sustained and high Ca^2+^ environment may accelerate the recruitment and differentiation of odontoblast-like cells and the initiation of mineral deposition. In contrast, BGM exhibited lowest Ca^2+^ release among all the materials yet still achieved favorable pulpal response. This successful outcome may be due to the presence of a distinct bioactive component in its composition. These material-dependent differences highlight that the presence of Ca^2+^ and other compositional factors might contribute to long term pulp healing and mineralization [37, 38]. However, extensive release of Ca^2+^ might be responsible for poor marginal integrity, which requires further investigations.

SBF is a physiological ionic composition that mimics the human blood plasma, providing an in vitro environment that promotes apatite formation and is widely used for bioactivity assessment [39]. It is generally accepted that the apatite-like layer formation in such body fluid facilitates mesenchymal cell recruitment by the bioceramic-based pulp capping materials, enabling highly active tissue repair or regeneration [40]. However, the extent and form of deposition varied among the materials. For instance, PMTA, BCR, and WPS showed more well-formed calcium-phosphate depositions compared to BGM. The surface of BCR and WPS developed well-formed, globular clusters of apatite, while PMTA presented a more granular deposition. This morphology significantly influences the bioactivity of the materials, where small particles absorb more protein, leading to further absorption of cells that can induce hard tissue regeneration [41]. Therefore, the globular morphology formed in the BCR and WPS groups can induce greater cell proliferation and mineralization. The authors speculated that such deposition might play an important role in creating a tight seal between the material and the dentinal walls, which is crucial for the success of most VPT treatments [42]. In addition, the calcium-rich phosphate deposits ability of these bioceramics materials might play a significant role in increasing the bioactivity and biocompatibility required for dental pulp repair [40]. In fact, EDX analysis revealed the presence of calcium and silicon, which are indicative of calcium silicate hydrates [43]. The ratio of Ca/P significantly affects the degree of bioactivity of the material. In this study, EDS analysis revealed significantly higher Ca/P ratios in the DW group, particularly in PMTA and WPS, compared to the SBF group. The SBF-immersed samples showed more balanced Ca/P ratios, consistent with apatite-like calcium phosphate deposition, which facilitates the nucleation and growth of an apatite layer and supports the material's bioactivity [44]. Previous studies reported that the Ca/P ratios of MTA-based materials had high ratios (18,27). These ratios were higher than the stoichiometric Ca/P ratio for hydroxyapatite (Ca/P = 1.67) [41]. Higher Ca/P ratios indicate calcium precipitation on the surface, which can lead to the desired bioactivity, biocompatibility, and hard tissue-induction abilities [41]. The Ca/P ratios obtained in this study were higher in all the groups than the stoichiometric ratio of hydroxyapatite. Therefore, it is likely that these materials would possess the abovementioned properties. Future studies involving longer immersion times or more sensitive structural characterization, such as FTIR or XRD, may help to confirm the precise nature of the mineral phase. On the other hand, although the tested materials immersed in DW exhibited no deposition after 28 days, they exhibited different microstructural features. The differences in microstructural features in the tested materials might be due to their compositional differences. As a result, the variations in composition could influence the dissolution behavior, ion release, and subsequent interaction with the surrounding environment [27]. These factors may ultimately affect the bioactivity and performance of the materials in clinical applications [27].

The in vivo findings provide critical insights into the biological responses and MTF abilities of the tested materials over varying time points [20, 21]. The present study revealed significant differences between the tested materials in terms of pulpal inflammation and MTF (*p* < 0.05). Initially, at days 1 and 3, mild inflammatory responses were observed in BCR and BGM, which might suggest their ability to limit acute inflammatory reactions compared to PMTA and WPS. This aligns with previous studies indicating that modified calcium silicate-based cements might exhibit less inflammatory responses [45], possibly due to their compositional differences. Although all the tested materials exhibited different inflammatory responses, which are beneficial and necessary for pulp healing and MTF, these inflammatory responses were limited and did not lead to extensive necrosis and cell apoptosis [46]. Additionally, the presence of bioactive ions such as calcium and silicon in tested BPC materials could promote a more favorable cellular response by enhancing biomineralization and reducing pulp irritation [3]. The thin layer of MTF was observed in the BCR group on day 3, although the exact reasons are yet to be elucidated. At day 7, a layer of MTF was observed across all groups, with homogeneity in the PMTA and BCR groups, possibly due to their effective stimulation of odontoblast-like cell differentiation and mineralization processes [47, 48]. Finally, on day 28, the BCR, BGM, and WPS groups demonstrated the absence of inflammation, providing a stable and favorable environment for long-term pulp healing. Notably, the superior performance of BCR, BGM, and WPS in achieving complete homogenous MTF emphasizes their potential as viable alternatives to traditional materials like PMTA.

In this present study, three different immunohistochemical markers were used. Nestin is a marker for newly differentiated odontoblast like cell identification, whereas osteopontin and DMP-1 are non-collagenous protein that indicate matrix mineralization and intra- and extra-cellular signaling molecules [49, 50]. The present study revealed that positive expression of Nestin was observed in all the groups except NC group at 28 days. Our observations showed clear expression of Nestin in both the cell bodies and the processes of cells lining the newly formed MTF. The observed Nestin expression in odontoblast-like cells is consistent with findings from previous studies [20, 21, 49]. DMP-1 expressions were observed at the exposure area in the tested group, indicating active odontoblastic differentiation and mineral deposition [2, 49]. These findings highlight the crucial role of material composition in regulating MTF and the cellular response [51]. Moreover, DMP-1 expression was observed within the complete MTF. This observation is consistent with previous studies [20, 21]. This might act as a signaling molecule and regulate odontoblasts-like cell differentiation, although it is well-known that DMP-1 also acts as a substrate or a scaffold for supporting pulpal cells to differentiate into odontoblast-like cells [20]. On the other hand, strong positive Osteopontin expression was observed after 3, 7, and 28 days, suggesting its role in the reparative process of exposed pulps, including the attraction of differentiating progenitors of newly generated odontoblast-like cells, attraction of macrophages for wound debridement, and stimulation of healing [49, 52]. Inflammatory cells such as macrophages, fibroblasts, and/or circulating molecules could be the origin of osteopontin, although this requires further study.

The present study has limitations. Firstly, it focused solely on the release of Ca^2+^. While calcium is a critical component for apatite formation and cellular signaling, the release kinetics of other important ions, such as silicon, phosphate, and strontium could provide a more comprehensive understanding of the materials interaction. Therefore, future studies should aim to quantify the release of these ions to better characterize the full bioactive potential. Another limitation of this study is that the immunohistochemical expression was assessed qualitatively. Future studies should incorporate quantitative analysis to better compare expression levels across experimental groups. The antimicrobial efficacy was not evaluated in this study. In addition, the tested materials were not evaluated in an inflamed dental pulp environment, which needs to be elucidated further. Tooth discoloration and mechanical properties are important parameters that can be evaluated in future studies.

## Conclusion

The present study demonstrated that all the BPC materials exhibited favorable bioactivity and biocompatibility with variable performance across parameters such as pH, Ca^2+^ release, inflammatory response, and mineralized tissue formation. Among the tested materials, BCR showed consistently high alkalinity, Ca^2+^ release, along with minimal inflammatory responses and early indications of mineralization. However, these early mineralization features were limited in extend and should be interpret with caution. While BPC materials may offer promising characteristics as a pulp capping, further studies are needed to confirm its clinical advantage over other materials.
